# An acoustic key to eight languages/dialects: Factor analyses of critical-band-filtered speech

**DOI:** 10.1038/srep42468

**Published:** 2017-02-15

**Authors:** Kazuo Ueda, Yoshitaka Nakajima

**Affiliations:** 1 Kyushu University, Department of Human Science/Research Center for Applied Perceptual Science, Fukuoka, 815-8540, Japan

## Abstract

The peripheral auditory system functions like a frequency analyser, often modelled as a bank of non-overlapping band-pass filters called critical bands; 20 bands are necessary for simulating frequency resolution of the ear within an ordinary frequency range of speech (up to 7,000 Hz). A far smaller number of filters seemed sufficient, however, to re-synthesise intelligible speech sentences with power fluctuations of the speech signals passing through them; nevertheless, the number and frequency ranges of the frequency bands for efficient speech communication are yet unknown. We derived four common frequency bands—covering approximately 50–540, 540–1,700, 1,700–3,300, and above 3,300 Hz—from factor analyses of spectral fluctuations in eight different spoken languages/dialects. The analyses robustly led to three factors common to all languages investigated—the *low & mid-high* factor related to the two separate frequency ranges of 50–540 and 1,700–3,300 Hz, the *mid-low* factor the range of 540–1,700 Hz, and the *high* factor the range above 3,300 Hz—in these different languages/dialects, suggesting a language universal.

Plomp and colleagues^1^,^2^,^3^,^4^ found that two acoustic principal components were enough to represent Dutch steady vowels. They extracted principal components from level profiles obtained from a bank of bandpass filters with bandwidths similar to those of critical bands, representing frequency-analysis properties of the auditory periphery, i.e., the basilar membrane^3^,^5^,^6^,^7^,^8^,^9^. They found a clear correspondence between the principal components and the conventional formant analyses^2^. In the present investigation, the principal-component-analysis (PCA) technique as pioneered by Plomp and his colleagues, and further pursued by Zahorian and Rothenberg^10^, was extended in two aspects: First, it was applied to a database^11^ of complete spoken sentences (58–200, depending on languages) rather than steady vowels, and second, the sentences were spoken in eight different languages/dialects, i.e., American English, British English, Cantonese, French, German, Japanese, Mandarin, and Spanish by 10–20 speakers in each language (Table 1; Supplementary Fig. S1 shows a block diagram of the analyses).

## Results

**Figure 1:**
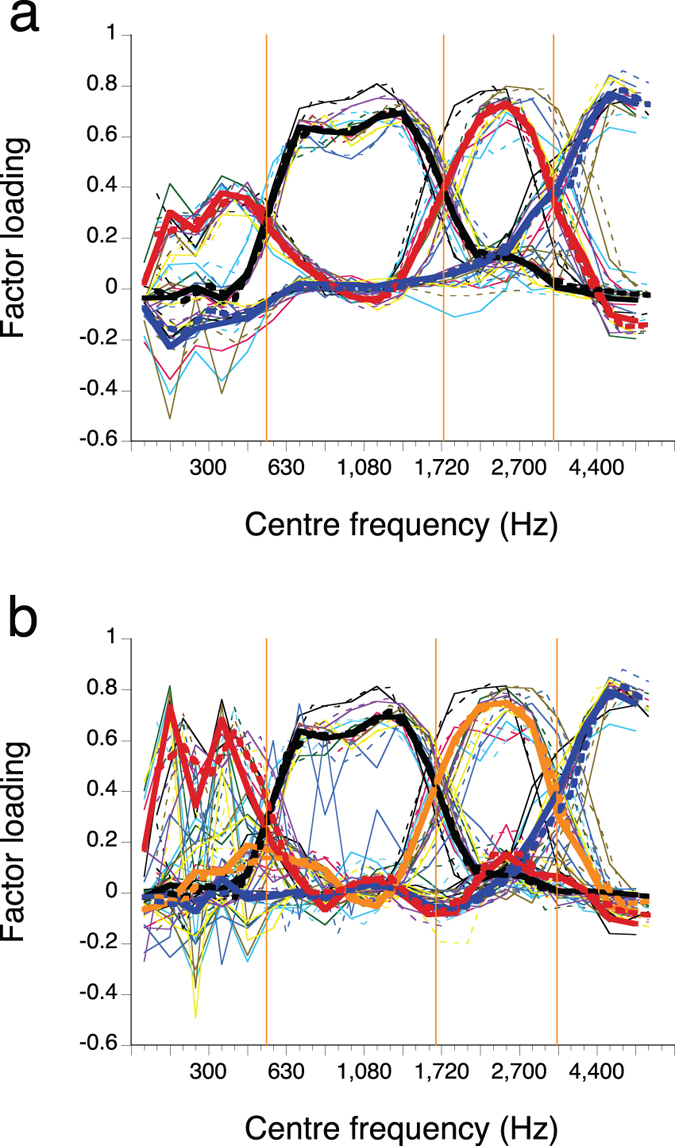
Factor loadings plotted against the centre frequency of critical bands. **(a)** Three-factor analysis. **(b)** Four-factor analysis. The thick lines represent factor loadings derived from the merged data across eight languages/dialects; the colours of the thick lines are to distinguish factors. The thin lines show the results of individual languages/dialects without distinguishing factors: American English (pink), British English (dark green), Cantonese (purple), French (sky blue), German (black), Japanese (blue), Mandarin (yellow), and Spanish (olive green). The broken lines are the counterparts of the solid lines of the same colours, using a filter-bank shifted up by half a critical bandwidth (Supplementary Table S1). The cumulative contributions ranged from 33–41% (**a**) and from 40–47% (**b**), depending on the analysed data set and the utilised filters. One division of the horizontal axis corresponds to 0.5 critical bandwidth, with the two sets of centre frequencies alternating. Orange vertical lines represent schematic frequency boundaries estimated from crossover frequencies of the curves.

Four blocks of critical bands, i.e., four frequency bands, consistently appeared in both the three- (Fig. 1a) and the four-factor (Fig. 1b) results—one of the factors obtained in the three-factor analysis was bimodal, thus both three- and four-factor analyses yielded four frequency bands. Two-, five-, and six-factor analyses gave rather obscure—inconsistent among the languages/dialects—results (Supplementary Fig. S2). The boundary frequencies dividing the whole frequency range into the four frequency bands are represented with the vertical orange lines in Fig. 1. Shifting the cut-off frequencies of the filter bank upwards by half a critical band (see Methods: Signal processing and analyses) had negligible effects on the three-factor results (Fig. 1a; compare the broken vs. continuous curves of the same colours). The three-factor results (Fig. 1a) exhibited greater agreement across the different languages/dialects than the four-factor results (Fig. 1b). The cumulative contributions, representing proportions of variance explained by the combinations of specified factors, were about 7% higher in the four-factor analysis (Fig. 1b), but the locations of the factor peaks were very similar comparing the three-factor with the four-factor analysis. The discrepancies between languages/dialects, observed in the lowest frequency band in the four-factor analysis, is likely to have been caused by the inclusion of samples spoken by speakers with relatively high fundamental frequency that could make frequency components too sparse in spectra. Including more than four factors resulted in cumulative contributions larger than 50%, however, the added factors were mainly consumed in capturing resolved harmonics in the low frequency region (Supplementary Fig. S2d,e), which was covered by a peak in the lower frequency side of the bimodal factor (low & mid-high factor) in the three-factor results. Thus, it seems to be optimal to take the three factor results for our present purpose, which is to find out the number and frequency ranges of the frequency bands for efficient speech communication.

## Discussion

It is worth noting that spoken sentences can be recognised even when they are conveyed only by power fluctuations of four frequency bands without any temporal fine structure, i.e., through noise-vocoded speech^12^,^13^,^14^,^15^,^16^,^17^,^18^. The number and location of these frequency bands (Fig. 1) is suggested both by the present physical analysis and by perceptual studies showing high intelligibility of noise-vocoded speech filtered into nearly the same^18^ or very similar^12^,^13^,^14^ frequency bands (Supplementary Audios S1 and S2, and Fig. S3). The four-band division must have some value in speech processing if it can be applied to several languages/dialects of different language families. Our own observation showed that the frequency boundaries or factors derived with the present statistical technique were suitable for synthesising noise-vocoded speech in Japanese^18^,^19^ and German^18^.

There seems a connection between the present frequency boundaries and the past results of speech-filtering investigations. The second boundary frequency, 1,700 Hz, was located near the centre of the range of the crossover frequency (typically from 1,550–1,900 Hz)^20^,^21^,^22^,^23^, which had been derived as a balancing point of intelligibility between highpass and lowpass filtering of speech. It is also to be noted that the frequency response of the telephone system is standardised to cover the range from 300–3,400 Hz. This frequency range covers at least a part of each frequency band in Fig. 1, presumably enabling the analogue telephone line to convey speech sounds all over the world with minimum cost and reasonable intelligibility.

We designated the factors obtained in the three-factor analysis as the *low & mid-high* factor, which appeared in two frequency ranges around 300 and around 2,200 Hz, the *mid-low* factor, which appeared around 1,100 Hz, and the *high* factor, which encompasses the range above 3,300 Hz. These factors appeared with surprising resemblance across the eight different languages/dialects of three different language families, and thus they are strong candidates for universal components of spoken languages/dialects, i.e., an acoustic language universal. An initial extension of the present analysis into infant utterances has been explored by a research team including the present authors^24^. One way to know how the factors relate to speech perception is to examine the correspondence between factor scores and phonemic categories. This line of investigation on speech sounds in British English has been started, as described in a separate paper.

## Methods

The following facts rationalise the use of the PCA-based technique in the present investigation. In order to recognise speech in quiet, it is not always necessary to fully utilise the frequency resolution properties of the basilar membrane. It is possible to accurately recognise speech consisting of power fluctuations in only four frequency bands (noise-vocoded speech^12^). Although this finding had been replicated in a number of studies^13^,^14^,^15^,^16^,^17^, the frequency cut-offs to create such frequency bands have not been derived from systematic research. One of the goals of the present study was to provide the characteristics of frequency channels that best represent the speech signal.

### Speech samples

Speech samples were extracted from a speech database^11^ (16-kHz sampling and 16-bit linear quantisation), upon the condition that the same set of sentences was spoken by all the speakers within each language/dialect. The samples were edited to eliminate irrelevant silent periods and noises. The details of the samples are shown in Table 1.

### Signal processing and analyses

Two banks (A and B) of 20 critical-band filters were constructed (Supplementary Table S1). Their centre frequencies ranged from 75–5,800 Hz (for A) and from 100–6,400 Hz (for B). Their overall passbands were 50–6,400 and 50–7,000 Hz, respectively. These two specific filter banks were made in order to check whether there was any artefact caused by cut-off frequencies in the analyses. The cut-off frequencies of each filter in bank A were determined according to Zwicker and Terhardt^6^, except for the lowest cut-off frequency (50 Hz). The cut-off frequencies in bank B were halfway shifted from those in bank A, except for the lowest cut-off frequency. All subsequent analyses were performed separately for these two filter banks. Each filter was constructed as a concatenate convolution of an upward frequency glide and its temporal reversal. Transition regions were 100 Hz wide, with out-of-band attenuations of 50–60 dB. Each filter output was squared, smoothed with a Gaussian window of σ = 5 (ms) which was equivalent to a lowpass filtering with a 45-Hz cut-off, and sampled at every millisecond. Because our analyses primarily focused on relatively slow movements of the vocal tract (amplitude envelopes) rather than fast movements of the vocal folds (temporal fine structure), power fluctuations were calculated by squaring and smoothing the filter outputs, in stead of using the outputs (amplitudes) themselves. Determining correlation coefficients for every possible combination of the power fluctuations yielded a correlation matrix for each data set. This matrix was fed into the PCA. That is, a correlation-based (normalised) analysis was selected, rather than a covariance-based one, in order to prevent the influence of unbalanced weighting between frequency bands of unequal power levels. After PCA was performed, the first 2–6 principal components were rotated with varimax rotation to yield the factors shown in Supplementary Fig. S2 (the terminology is based on convention).

## Additional Information

**How to cite this article**: Ueda, K. and Nakajima, Y. An acoustic key to eight languages/dialects: Factor analyses of critical-band-filtered speech. *Sci. Rep.* **7**, 42468; doi: 10.1038/srep42468 (2017).

**Publisher's note:** Springer Nature remains neutral with regard to jurisdictional claims in published maps and institutional affiliations.

## Supplementary Material

Supplementary Information

Supplementary Audio S1

Supplementary Audio S2

## Figures and Tables

**Table 1:** Analysed speech samples.

Languages/dialects	Number of sentences	Number of speakers		Overall duration of utterances (s)	Mean duration per utterance (s)
		Female	Male		
American English	86	10	10	4,123.2	2.4
British English	200	5	5	4,038.5	2.0
Cantonese	58	5	5	1,131.7	2.0
French	200	5	5	3,533.2	1.8
German	200	5	5	3,707.0	1.9
Japanese	200	5	5	5,041.3	2.5
Mandarin	78	5	5	1,834.9	2.4
Spanish	136	5	5	2,918.1	2.1
Total	1,158	45	45	26,327.8	2.1

Speech samples were extracted from the database^11^.

## References

[b1] Plomp R., Pols L. C. W. & van de Geer J. P. Dimensional analysis of vowel spectra. J. Acoust. Soc. Am. 41, 707–712 (1967).

[b2] Pols L. C. W., Tromp H. R. C. & Plomp R. Frequency analysis of Dutch vowels from 50 male speakers. J. Acoust. Soc. Am. 53, 1093–1101 (1973). 4697809 10.1121/1.1913429

[b3] Plomp R. Aspects of Tone Sensation: A Psychophysical Study (Academic Press, London, 1976).

[b4] Plomp R. The Intelligent Ear: On the Nature of Sound Perception (Lawrence Erlbaum, Mahwah, New Jersey, 2002).

[b5] Fletcher H. Auditory patterns. Rev. Mod. Phys. 12, 47–65 (1940).

[b6] Zwicker E. & Terhardt E. Analytical expressions for critical-band rate and critical bandwidth as a function of frequency. J. Acoust. Soc. Am. 68, 1523–1525 (1980).

[b7] Greenwood D. D. A cochlear frequency-position function for several species–29 years later. J. Acoust. Soc. Am. 87, 2592–2605 (1990). 2373794 10.1121/1.399052

[b8] Schneider B. A., Morrongiello B. A. & Trehub S. E. Size of critical band in infants, children, and adults. J. Exp. Psychol. Human Percept. Perf. 16, 642–652 (1990). 10.1037//0096-1523.16.3.642 2144577

[b9] Unoki M., Irino T., Glasberg B., Moore B. C. J. & Patterson R. D. Comparison of the roex and gammachirp filters as representations of the auditory filter. J. Acoust. Soc. Am. 120, 1474–1492 (2006). 17004470 10.1121/1.2228539 PMC2825387

[b10] Zahorian S. A. & Rothenberg M. Principal-components analysis for low-redundancy encoding of speech spectra. J. Acoust. Soc. Am. 69, 832–845 (1981).

[b11] NTT-AT. Multi-lingual speech database 2002 (2002).

[b12] Shannon R. V., Zeng F.-G., Kamath V., Wygonski J. & Ekelid M. Speech recognition with primarily temporal cues. Science 270, 303–304 (1995). 7569981 10.1126/science.270.5234.303

[b13] Dorman M. F., Loizou P. C. & Rainey D. Speech intelligibility as a function of the number of channels of stimulation for signal processors using sine-wave and noise-band outputs. J. Acoust. Soc. Am. 102, 2403–2411 (1997). 9348698 10.1121/1.419603

[b14] Smith Z. M., Delgutte B. & Oxenham A. J. Chimaeric sounds reveal dichotomies in auditory perception. Nature 416, 87–90 (2002). 11882898 10.1038/416087a PMC2268248

[b15] Riquimaroux H. Perception of noise-vocoded speech sounds: Sentences, words, accents and melodies. Acoust. Sci. Tech. 27, 325–331 (2006).

[b16] Sheldon S., Pichora-Fuller M. K. & Schneider B. A. Effect of age, presentation method, and learning on identification of noise-vocoded words. J. Acoust. Soc. Am. 123, 476–488 (2008). 18177175 10.1121/1.2805676

[b17] Roberts B., Summers R. J. & Bailey P. J. The intelligibility of noise-vocoded speech: spectral information available from across-channel comparison of amplitude envelopes. Proc. Royal Soc. B 278, 1595–1600 (2011). 10.1098/rspb.2010.1554 PMC3081737 21068039

[b18] Ellermeier W., Kattner F., Ueda K., Doumoto K. & Nakajima Y. Memory disruption by irrelevant noise-vocoded speech: Effects of native language and the number of frequency bands. J. Acoust. Soc. Am. 138, 1561–1569 (2015). 26428793 10.1121/1.4928954

[b19] Kishida T., Nakajima Y., Ueda K. & Remijn G. Three factors are critical in order to synthesize intelligible noise-vocoded Japanese speech. Frontiers in Psychology 7 (2016). 10.3389/fpsyg.2016.00517 PMC4845253 27199790

[b20] French N. R. & Steinberg J. C. Factors governing the intelligibility of speech sounds. J. Acoust. Soc. Am. 19, 90–119 (1947).

[b21] Hirsh I. J., Reynolds E. G. & Joseph M. Intelligibility of different speech materials. J. Acoust. Soc. Am. 26, 530–538 (1954).

[b22] Miller G. A. & Nicely P. E. An analysis of perceptual confusions among some English consonants. J. Acoust. Soc. Am. 27, 338–352 (1955).

[b23] Studebaker G. A., Pavlovic C. V. & Sherbecoe R. L. A frequency importance function for continuous discourse. J. Acoust. Soc. Am. 81, 1130–1138 (1987). 3571730 10.1121/1.394633

[b24] Yamashita Y. . Acoustic analyses of speech sounds and rhythms in Japanese- and English-learning infants. Frontiers in Psychology 4, 1–10 (2013). 23450824 10.3389/fpsyg.2013.00057 PMC3584442

