# Supplementary material for: An acoustic key to eight languages/dialects: Factor analyses of critical-band-filtered speech
Source: Sci Rep. 2017 Feb 15;7:42468. doi: 10.1038/srep42468 (PMC5309770; doi:10.1038/srep42468)
Supplement: Supplementary Information [file srep42468-s1.pdf]

Supplementary Information
An acoustic key to eight languages/dialects: Factor analyses of critical-band-filtered speech

Kazuo Ueda[1*] **& Yoshitaka Nakajima**[1*]

[1]Kyushu University, Department of Human Science/Research Center for Applied Perceptual Science, Fukuoka, 815-8540, Japan
*email to K.U.: ueda@design.kyushu-u.ac.jp; email to Y.N.: nakajima@design.kyushu-u.ac.jp

Table of Contents

Supplementary Figures S1–S3
Supplementary Table S1
Supplementary Audios S1 and S2
References

Supplementary Figures

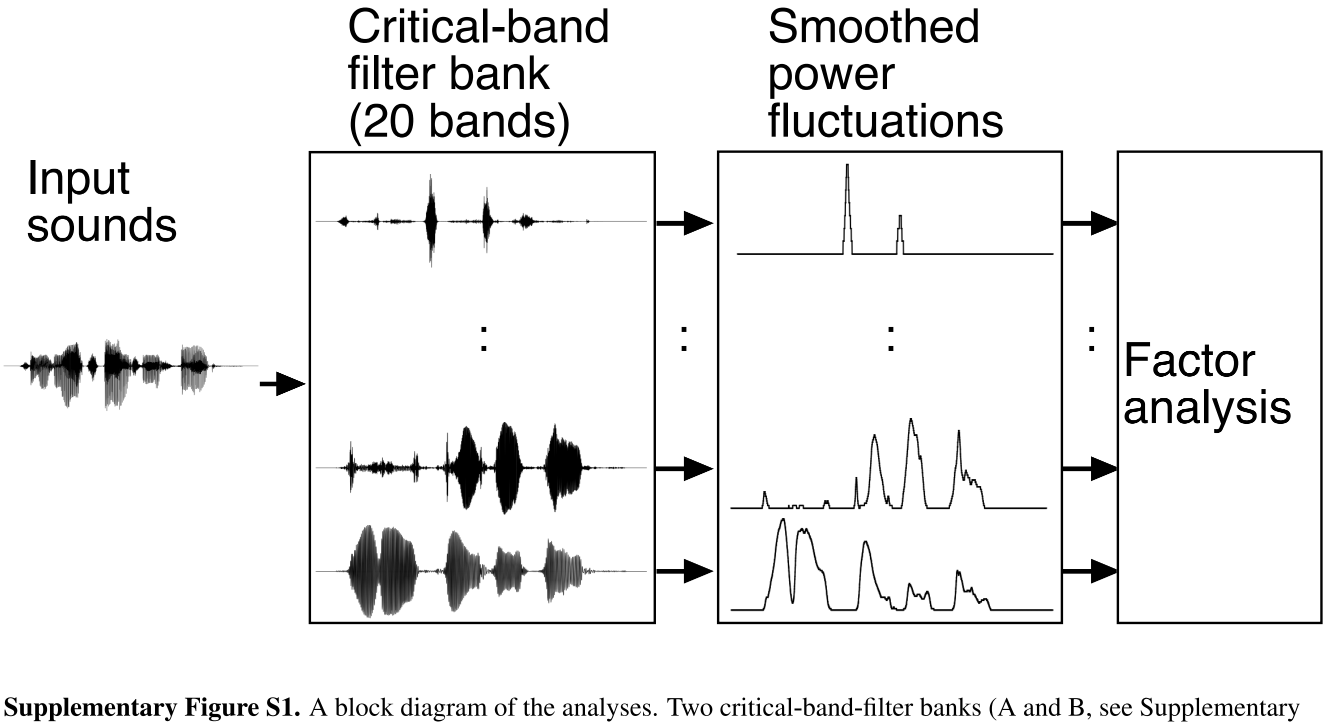

Supplementary Figure S1. A block diagram of the analyses. Two critical-band-filter banks (A and B, see Supplementary Table S1 online, for details) were used. Each filter output was squared and smoothed to obtain a power fluctuation. Factor analyses were based on correlation coefficients between the concurrent power fluctuations. Principal components analyses were applied to the correlation coefficient matrices, and then varimax rotation was applied to the first 2–6 components.

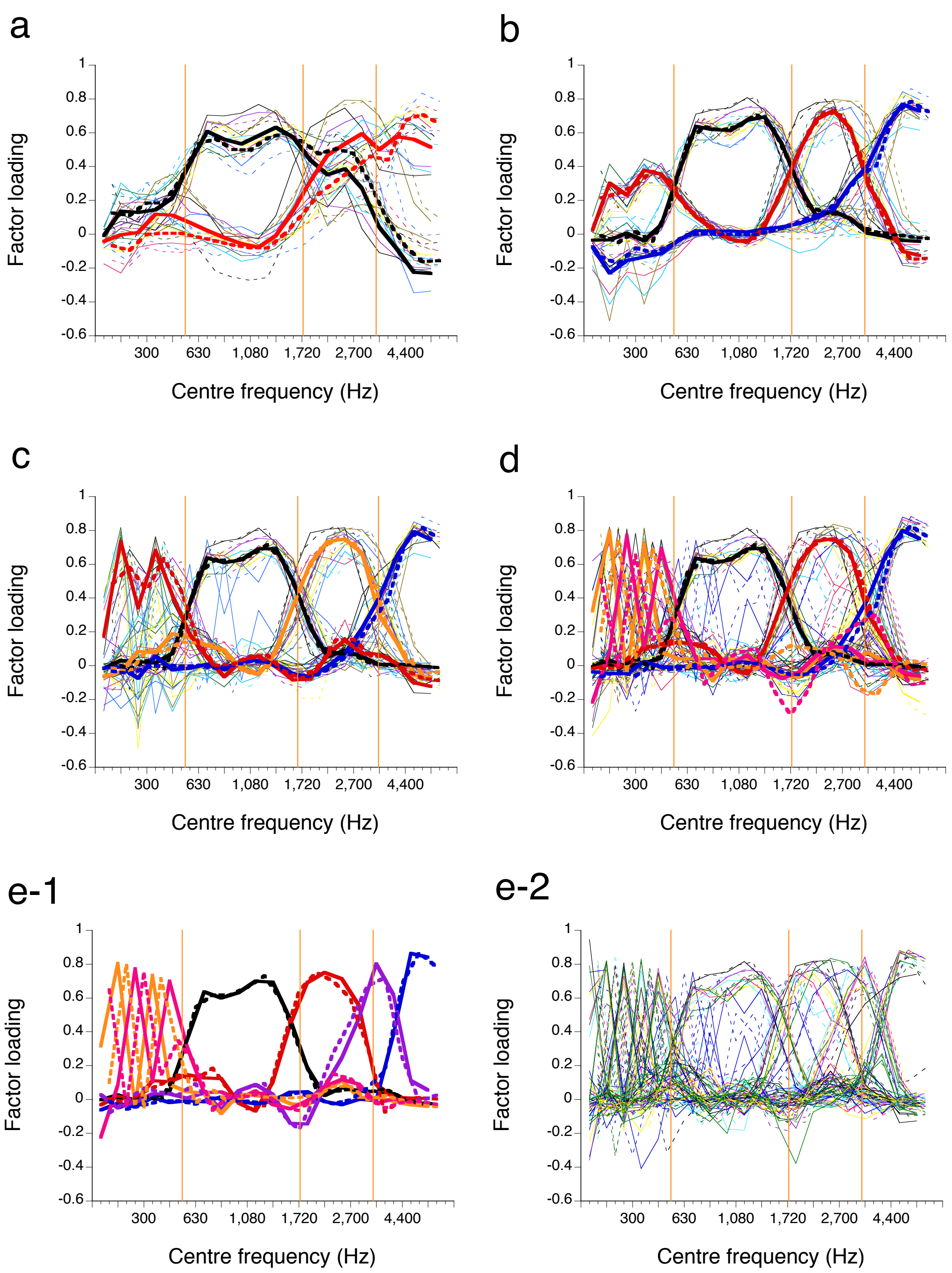

Supplementary Figure S2. Factor loadings plotted against the centre frequency of critical bands. (**a**) Two-factor analysis. (**b**) Three-factor analysis. (**c**) Four-factor analysis. (**d**) Five-factor analysis. (**e**) Six-factor analysis, divided into two figures, i.e., **e-1** and **e-2**, because of a technical reason. Panels (b) and (c) are the panels (a) and (b) in Fig. 1, respectively. The thick lines represent factor loadings derived from the merged data across eight languages/dialects; the colours of the thick lines are to distinguish factors. The thin lines show the results of individual languages/dialects without distinguishing factors: American English (pink), British English (dark green), Cantonese (purple), French (sky blue), German (black), Japanese (blue), Mandarin (yellow), and Spanish (olive green). The broken lines are the counterparts of the solid lines of the same colours, using a filter-bank shifted up by half a critical-band width. The cumulative contributions were ranged from 26–31% (**a**), from 33–41% (**b**), from 40–47% (**c**), from 46–53% (**d**), and from 51–57% (**e**), depending on the analysed data set and the utilised filters. One division of the horizontal axis corresponds to 0.5 critical-band width, with the two sets of centre frequencies alternating. Orange vertical lines represent schematic frequency boundaries estimated from crossover frequencies of the curves in the three-factor results (Fig. 1a) as a reference, except in the panel (**c**), where the orange vertical lines represent boundaries of the four-factor results (Fig. 1b).

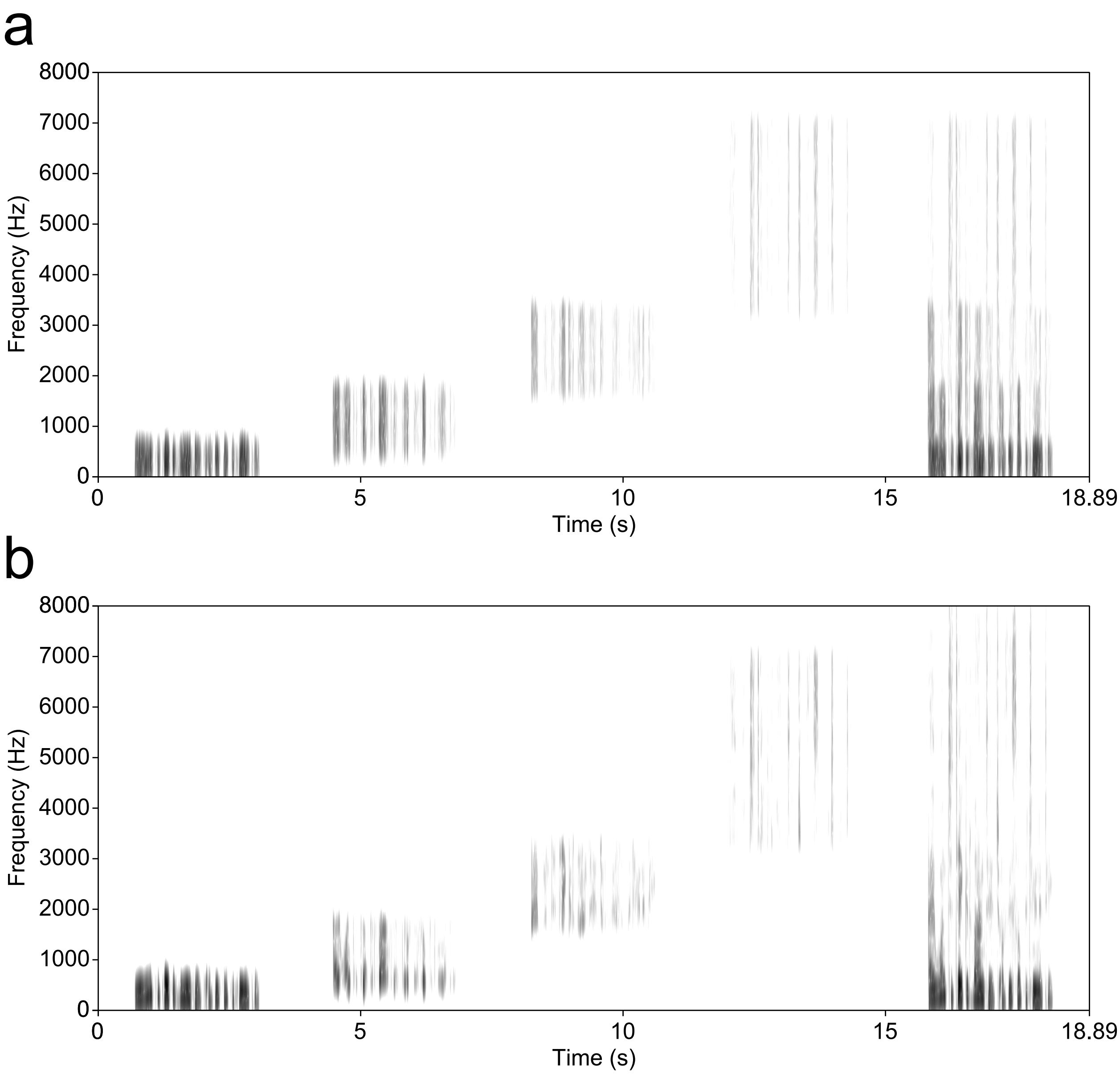

Supplementary Figure S3. Spectrogram of Supplementary Audios S1 (**a**) and S2 (**b**) online. See the caption of Audios S1 and S2 for detail.

Supplementary Table

Supplementary Table S1. Critical-band-filter settings. The centre frequencies and the passbands are expressed in Hz. The frequencies in the bank A were adopted from Zwicker and Terhardt[1], except altering the lowest cut-off frequency from 0 to 50 Hz. The centre frequencies of the filters in the bank B were placed at the upper cut-off frequencies of the corresponding filters in the bank A.

Band	Bank A		Bank B	
number	Centre frequency	Passband	Centre frequency	Passband
1	75	50–100	100	50–150
2	150	100–200	200	150–250
3	250	200–300	300	250–350
4	350	300–400	400	350–450
5	450	400–510	510	450–570
6	570	510–630	630	570–700
7	700	630–770	770	700–840
8	840	770–920	920	840–1,000
9	1,000	920–1,080	1,080	1,000–1,170
10	1,170	1,080–1,270	1,270	1,170–1,370
11	1,370	1,270–1,480	1,480	1,370–1,600
12	1,600	1,480–1,720	1,720	1,600–1,850
13	1,850	1,720–2,000	2,000	1,850–2,150
14	2,150	2,000–2,320	2,320	2,150–2,500
15	2,500	2,320–2,700	2,700	2,500–2,900
16	2,900	2,700–3,150	3,150	2,900–3,400
17	3,400	3,150–3,700	3,700	3,400–4,000
18	4,000	3,700–4,400	4,400	4,000–4,800
19	4,800	4,400–5,300	5,300	4,800–5,800
20	5,800	5,300-6,400	6,400	5,800-7,000

Supplementary Audios

Supplementary Audio S1. Four-band noise-vocoded speech. Each frequency band, i.e., 50–540, 540–1,700, 1,700–3,300, and 3,300–7,000 Hz, of noise-vocoded speech is presented one by one from the lowest band to the highest. Finally, all frequency bands are integrated. The original speech sample is presented in Supplementary Audio S2. The modification was granted permission by NTT-AT.

Supplementary Audio S2. The original speech sentence ("I want to take advantage of this opportunity.") spoken by a female British English speaker, which was taken from NTT-AT, Multi-lingual speech database 2002[2]. The same one-by-one bandpass filtering (but not noise-vocoding) as in Supplementary Audio S1 online was applied for the sake of comparison. The modification was granted permission by NTT-AT.

References

1. Zwicker, E. & Terhardt, E. Analytical expressions for critical-band rate and critical bandwidth as a function of frequency. *J. Acoust. Soc. Am.* **68**, 1523–1525 (1980).

2. NTT-AT. Multi-lingual speech database 2002 (2002).
